# Supplementary material for: The activating receptor NKG2D is an anti-fungal pattern recognition receptor
Source: Nat Commun. 2024 Oct 7;15:8664. doi: 10.1038/s41467-024-52913-2 (PMC11458907; doi:10.1038/s41467-024-52913-2)
Supplement: Supplementary file 1 — Supplementary Information [file 41467_2024_52913_MOESM1_ESM.pdf]

Supplemental Figure 1

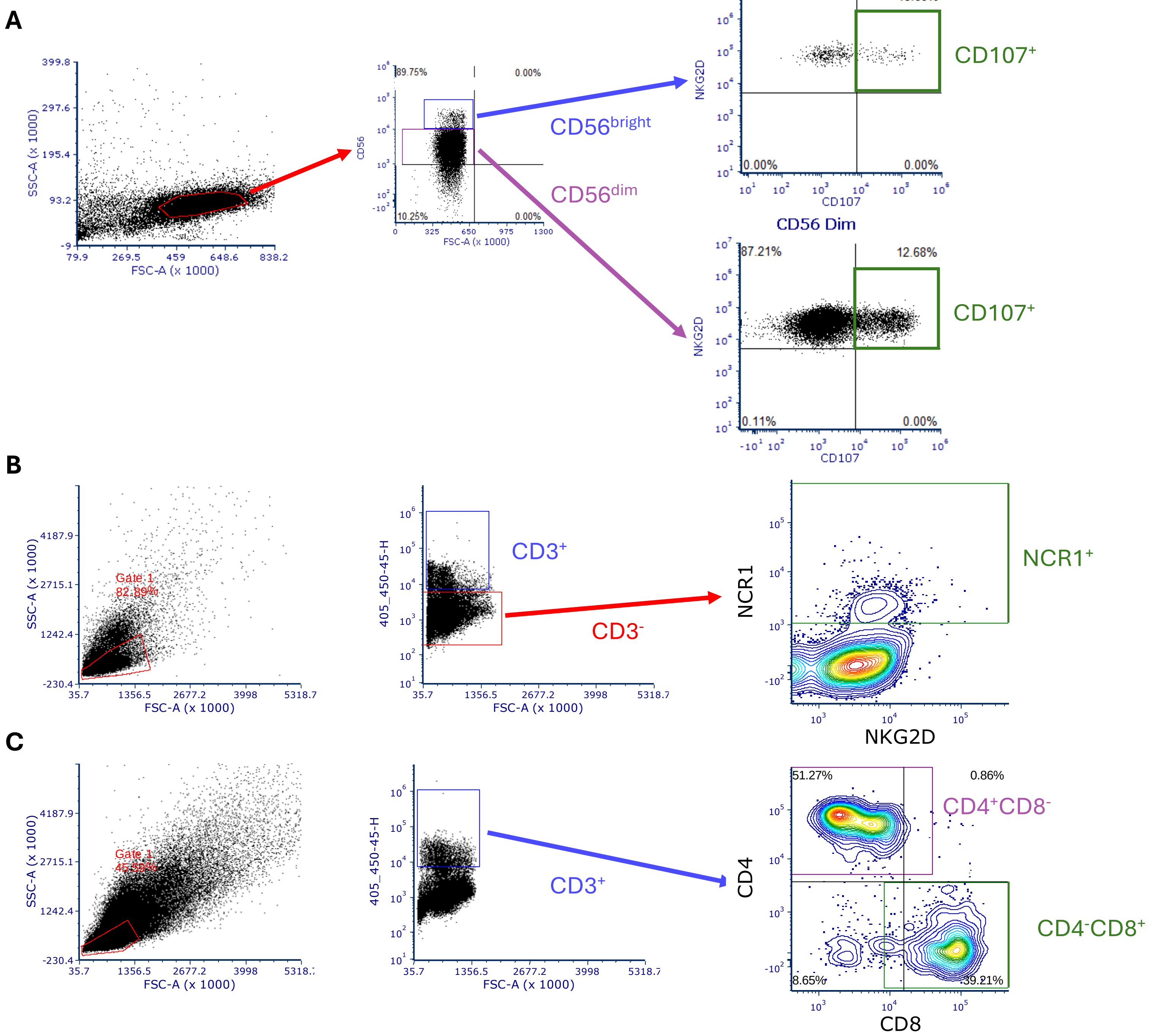

Supplementary figure 1, Gating strategy for flow cytometry experiments. related to figures 1 and 4.

A. Gating strategy of one representative staining of fresh human NK cells divided into CD56dim and CD56 bright populations and used for fig. 1H+I (a representative image of NK co-incubated with *C. albicans* is presented). **B.** Gating strategy of one representative staining of NK cells isolated from the blood of a *C. albicans* infected mouse used for fig. 4D-F. **C.** Gating strategy of one representative staining of T cells isolated from the blood of an uninfected mouse used for fig. 4D-F.

Supplemental Figure 2

A

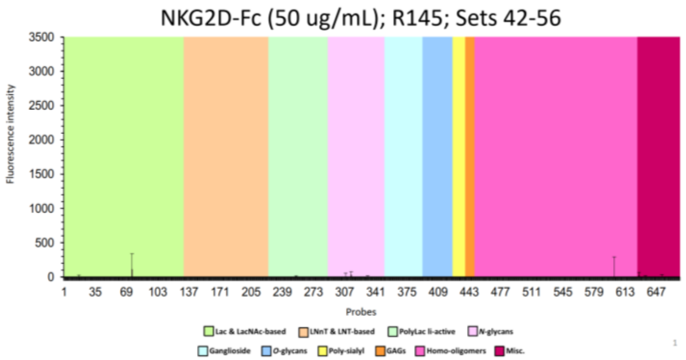

B

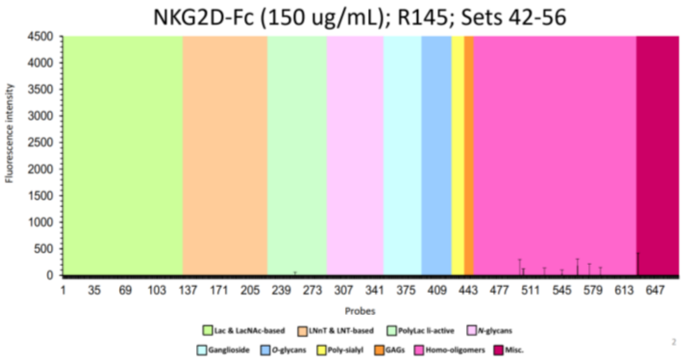

Supplemental figure 2. Glycan array scan for NKG2D-Ig binding partners.

A+B. Glycan microarray screening analyses of NKG2D-Ig, as a protein-antibody precomplex together with biotinylated anti-human IgG. Two different concentrations of protein-antibody complexes were tested: 50ug/ml (A) and 150ug/ml (B). The 672 lipid-linked probes are grouped into families as described in the legend under each graph.

The full list of glycan probes, their sequences, and binding scores are given as supplemental Dataset 1.

Supplemental Figure 3

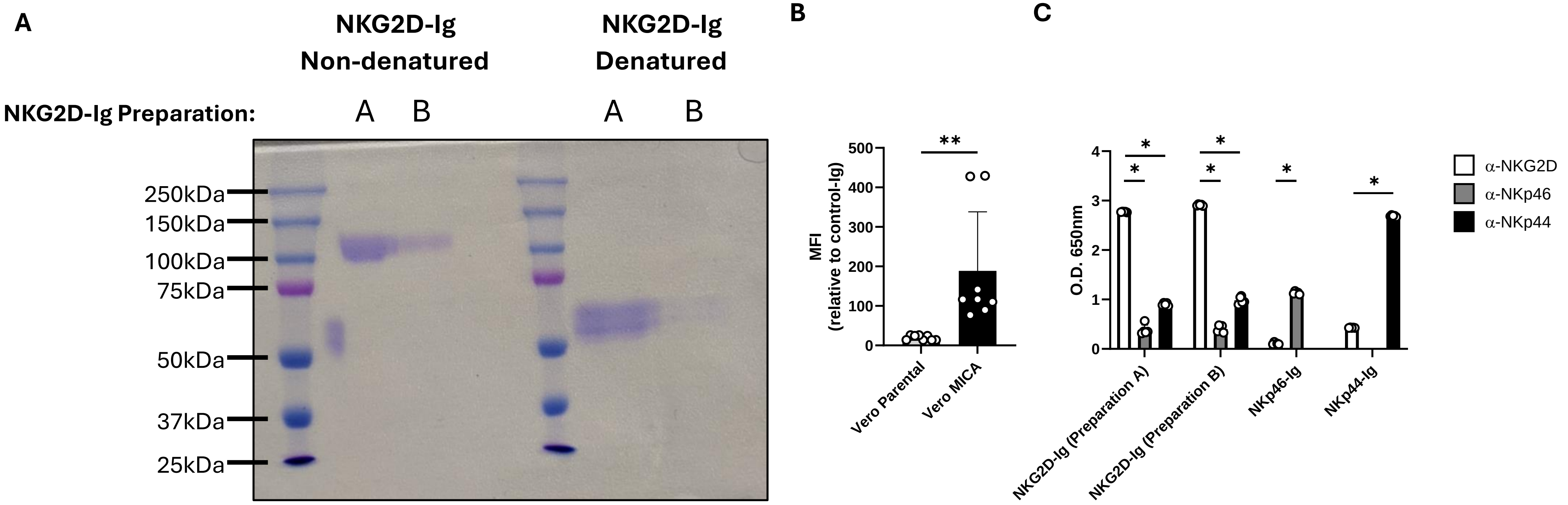

Supplemental figure 3. Ig fusion protein validation. Related to the methods section regarding Fc fusion protein generation

A. Coomassie staining of two independent preparations of NKG2D-Ig used in this study, after gel electrophoresis under reducing and non-reducing conditions**. B.** Quantification of Ig-fusion protein staining followed by flow cytometry of two cell lines: A parental line of Vero cells (white bar) and a daughter line in which we exogenously expressed the NKG2D ligand MICA (Vero MICA, black bar). Presented are relative MFI values of the NKG2D-Ig staining signal relative to staining with control-Ig. n=8. **C.** ELISA of plate-bound Ig fusion proteins (two independent preparations of NKG2D-Ig, a preparation of NKp46-Ig and a preparation of NKp44-Ig) stained with various antibodies to validate their identity. The antibodies used were α-NKG2D (white bars), α-NKp46 (grey bars) and α-NKp44 (black bars). Data for B+C is presented as mean±SEM. Significance was tested using Wilcoxon matched-pairs signed rank test (B) or multiple unpaired T-tests corrected for multiple comparisons using the FDR approach. * = $p < 0.05$, ** = $p < 0.01$.
