# Supplementary material for: The activating receptor NKG2D is an anti-fungal pattern recognition receptor
Source: Nat Commun. 2024 Oct 7;15:8664. doi: 10.1038/s41467-024-52913-2 (PMC11458907; doi:10.1038/s41467-024-52913-2)
Supplement: Supplementary file 3 — Description of Additional Supplementary Files [file 41467_2024_52913_MOESM3_ESM.pdf]

Description of Additional Supplementary Files

File Name: Supplementary Data 1
Description: Table with all commercial antibodies used in this study, and the information about them.
